# Study on re-entrant hierarchical honeycombs in-plane impact

**DOI:** 10.1038/s41598-023-48356-2

**Published:** 2023-12-05

**Authors:** Jinming Lian, Lei Xu, Donggao Wu, Zhenqing Wang

**Affiliations:** College of Aerospace and Civil Engineering, Harbin Engineering University, Harbin, 150001 China

**Keywords:** Mechanical engineering, Structural materials

## Abstract

The introduction of hierarchical structure in cell materials can further improve their energy absorption effect, and negative Poisson's ratio materials have excellent energy absorption capacity and special deformation mode. In this paper, augmented double arrow honeycomb structures is introduced into the re-entrant honeycomb with negative Poisson's ratio as a substructure (RHA) to improve the mechanical properties of the first-order re-entrant honeycomb and enhance the energy absorption effect of the structure. The analytical formula of the collapse stress of honeycomb under quasi-static compression was derived by the two-scale method. The failure stress of RHA under different relative densities and impact velocities is discussed, and the analytical formula of RHA stress in dynamic crushing is derived by combining momentum conservation. Due to the special substructure, the secondary honeycomb discussed in this paper has two plateau periods. In this paper, the second plateau stress of the honeycomb structure is calculated innovatively. The numerical simulation results show that the collapse stress of RHA in the first plateau period is similar to that of the first-order re-entrant honeycomb, and the collapse stress in the second plateau period is increased by 332%. The research in this paper shows that the honeycomb with the second plateau period has a better energy absorption effect, which is an effective strategy for improving the energy absorption effect of the honeycomb. It can be further explored to improve the impact resistance of the honeycomb.

## Introduction

The honeycomb structure is widely used in aerospace, construction, transportation, and other fields due to its excellent mechanical properties and excellent thermal conductivity^1–5^. There are also a large number of natural cell materials in nature, such as wood and bone, which are light in weight and generally have good mechanical properties. At present, the research on bionic honeycomb materials is also very hot. Zhang et al.^6^ studied the effects of honeycomb size and forming method on the mechanical properties of honeycomb panels, and found that the trabecular honeycomb core structure of beetle sheath-wing panels can double the compressive strength of honeycomb panels. Chen et al.^7^ designed a new type of single-sided bonded honeycomb panel by using the compression performance of the upper and lower honeycomb panels, and verified the effectiveness of the manufacturing method of the honeycomb bionic laminated structure for the first time through experiments. Liu et al.^8^ reported a novel bionic three-dimensional mesoporous cathode with a 'swarm-filled honeycomb' structure, which increased the maximum active material archiving fraction of the opal template to about 90% before pinching off.

Generally speaking, honeycomb structure is also a kind of hierarchical structure. The definition of hierarchical structure is as follows: continuous solid is a zero-order structure, and honeycomb structure was once considered as a first-order structure (such as foam material and traditional honeycomb material). The application of first-order honeycomb materials has been very mature and is widely used in aviation and construction. Based on the honeycomb fracture theory of hexagonal honeycomb in low-velocity impact revealed by numerical simulation, Hu et al.^9^ established an analytical model of compressive strength and support end stress of honeycomb with impact velocity, honeycomb size, honeycomb angle and mechanical properties of the substrate. Cricri et al.^10^ studied the stress characteristics of regular hexagonal honeycombs in the general plane through experiments and numerical calculations. Wu et al.^11^ conducted a series of experimental studies on the compressive strength of six kinds of aluminum honeycombs under out-of-plane and action. Mukhopadhyay et al.^12^ proposed a concept based on mechanics to study the frequency dependence of in-plane elastic modulus of lattice materials.

For the second-order honeycomb, because the cell wall is replaced by the first-order structure, not only does the main structure deform to absorb energy, but also the substructure changes to absorb the damage caused by the impact. Qiao et al.^13^ studied the in-plane uniaxial collapse response of two-stage layered honeycombs (i.e., regular hexagonal honeycombs, which are composed of equilateral triangular honeycombs). The failure modes of quasi-static crushing and dynamic impact in two directions were discussed by a finite element simulation system, and a two-scale method was proposed to obtain the analytical expression of quasi-static collapse stress of layered honeycombs in two directions. Song et al.^14^ introduced the augmented double-arrowhead honeycomb as a substructure into the hexagonal honeycomb. Through finite element simulation, the failure stress of the layered honeycomb in the quasi-static and dynamic crushing directions was analyzed. It is proved that the second-order layered honeycomb (SHH) has better collapse stress than the first-order hexagonal honeycomb (FHH) and the augmented double-arrowhead honeycomb (ADAH). Sun et al.^15^ proposed an anisotropic multi-functional layered honeycomb structure, and analyzed the in-plane stiffness of the structure through Euler beam theory. Zhang et al.^16^ constructed a sub-similar hexagonal layered honeycomb with fractal characteristics, and carried out parametric studies to explore strategies to improve out-of-plane crashworthiness by changing material distribution. Wu et al.^17^ proposed a new layered circular node quadrilateral honeycomb structure. Based on the simplified super folding element theory, the analytical solution of the crushing performance of the layered honeycomb was obtained. Yin et al.^18^ compared three different hierarchical honeycombs and confirmed that triangular honeycombs have the best crushing performance. Xu et al.^19^ adopted a new self-similar layered hexagonal column to improve the crashworthiness of the vehicle. Fang et al.^20^ replaced the side of the hexagon with a smaller hexagon, and applied a special institutional level to the honeycomb.

In recent years, there has been more and more studies on negative Poisson's ratio honeycomb materials. Negative Poisson's ratio materials have unique mechanical properties and good energy absorption capacity due to their non-plastic structure. Tan et al.^21^ combined the characteristics of deformed structures and layered honeycombs, and proposed two kinds of re-entrant honeycombs with regular hexagon substructure (RHH) and equilateral triangle substructure (RHT) instead of re-entrant honeycomb cell walls. The crashworthiness of the honeycomb was studied, and the plateau stress of the cross-section on x and y was derived by the two-scale method. Zhang, An and Qiao et al.^22–24^ found a new type of negative Poisson's ratio structure, verified the structure and evaluated the mechanical properties of the structure. Ma et al.^25^ established the nonlinear constitutive relation of honeycomb structure under the condition of large deformation, which provides a theoretical basis for the study of anechoic honeycomb sandwich structure. Based on the isogeometric analysis and refined shear deformation theory, Liu et al.^26^ established the discrete equilibrium equation of sandwich plates. Yang et al.^27^ explored the substitution effect of two honeycomb corrugated spring structures with different negative Poisson's ratios in space deployable structures. Yang et al.^28^ found that the size of the cell structure is the main factor affecting the equivalent Poisson's ratio and equivalent elastic modulus of the star-shaped three-dimensional negative Poisson's ratio composite structure. Xiao et al.^29^ have studied the influence of gradient arrangement and impact compression speed on the deformation failure mode and dynamic response curve of honeycomb structure. The negative gradient honeycomb structure has the best energy absorption effect.

The honeycomb structure generally undergoes two deformation modes when it is impacted, one is the rotation deformation of the side cell wall, and the other is the compression deformation of the side cell wall. For the negative Poisson's ratio honeycomb, the deformation mode is basically the same as that of the ordinary honeycomb. However, due to the particularity of the structure, when the negative Poisson's ratio honeycomb receives the impact, the honeycomb structure shrinks internally with the occurrence of the impact. This special deformation mode has attracted wide attention. This paper explores a negative Poisson's ratio honeycomb under a new substructure.

In this paper, the augmented double arrow honeycomb is embedded as a substructure (ADAH) into the re-entrant honeycomb cell wall to form a new second-order honeycomb structure, which I call RHA. Comparing the second-order honeycomb structure RHA with the traditional re-entrant honeycomb RH, it is verified that RHA has better collapse stress than RH. At present, the evaluation of the energy absorption effect of the honeycomb structure is based on the platform stress, but the second platform stress of the deformation of the honeycomb structure has not been paid attention to. This paper creatively calculates the second platform stress of the honeycomb, which provides a new strategy for improving the energy absorption effect of the honeycomb structure. The paper is organized as follows. In the "Finite element modeling of hierarchical honeycomb" section, we introduce the materials and structures of second-order honeycombs and the methods, and verify the results of finite element analysis with the results of Qiao et al.^13^. In the "Quasi-static collapse response" section, the quasi-static collapse response of honeycomb in the x and y directions is studied by the finite element simulation method, and the quasi-static collapse stresses of the hierarchical honeycombs in two directions is derived by finite element deformation mode. In the "Dynamic collapse response" section, we discuss the impact response at different speeds. In the "Concluding remarks" section, we give some conclusions.

## Finite element modeling of hierarchical honeycomb

**Figure 1:**
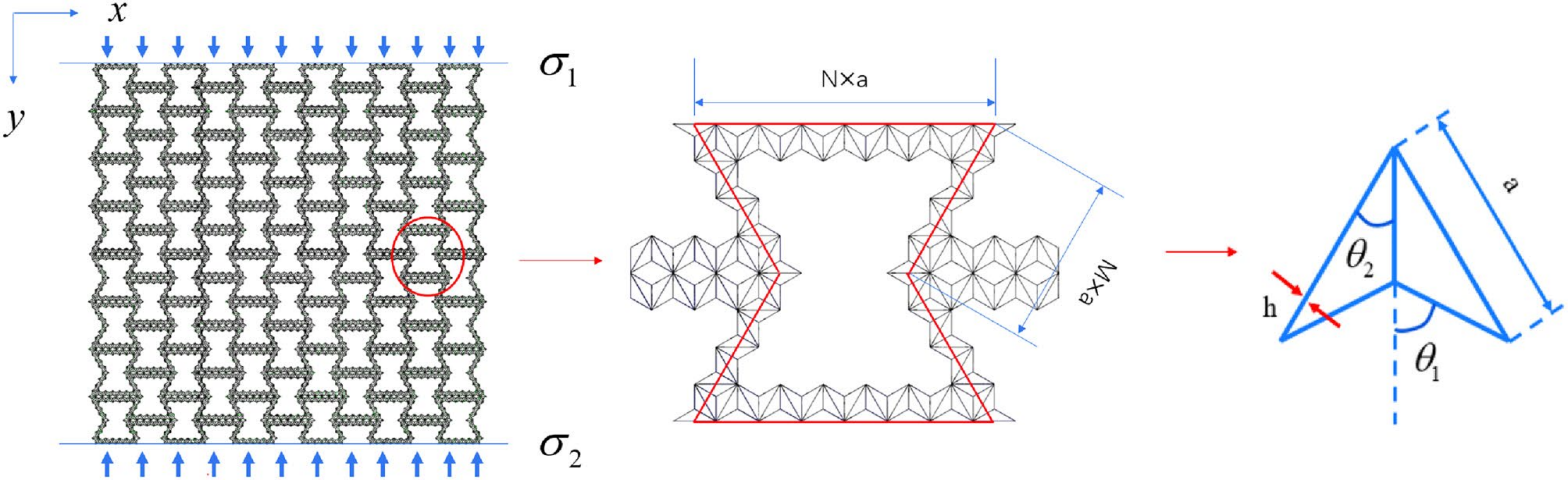
RHA and its cell diagram under impact load.

As discussed in the "Introduction" section, the appropriate introduction of a hierarchical structure in the honeycomb structure can further improve its mechanical properties. In this paper, the augmented double arrow honeycomb (ADAH) is introduced into the re-entrant honeycomb cell wall as a substructure to form a new type of second-order honeycomb structure, which I call RHA. Figure 1 is the RHA model under uniaxial compression. The macroscopic structure of the RHA is a re-entrant honeycomb, and its cell wall is composed of isosceles triangles. Here a is the length of the isosceles triangle’s long side, and b is the out-of-plane thickness. The length of the horizontal edge is N × a, and the length of the inclined edge is M × a. N is the number of substructures on the horizontal edge, and M is the number of substructures on the inclined edge,as shown in Fig. 1.

Assuming that the thickness of the substructure cell wall is uniform and h is the thickness of the RHA microscopic cell wall, the relative density is

1 $$\overline{\rho } = h \cdot \frac{8a(N + M + a) + 4\sqrt 3 a(2M + 2N + 1)}{{\frac{\sqrt 3 }{3}a^{2} (2N - M + 1)(3 + 3M)}}$$

Due to the development of industry, higher requirements are put forward for modern materials, one is to have good mechanical properties, and the other is to be economical. Aluminum has excellent mechanical properties and low cost. Therefore, the matrix material used in this paper is aluminum, In all models, the solid material constituting the RHAs is assumed to be rate independent elastically perfectly plastic with mass densityρ = 2700 kg/m^3^, elastic modulus E = 70GPa, Poisson's ratio ν = 0.3, and yield stress σ = 110 MPa.

**Figure 2:**
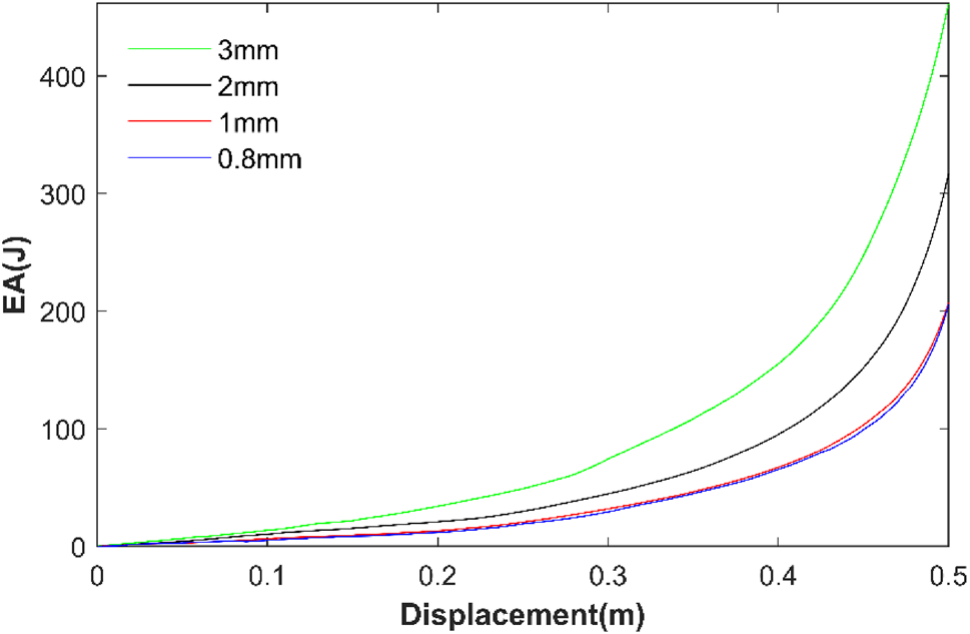
Convergence analysis of element size.

The finite element software is Abaqus commercial software. In the FE model, 8 × 11 cells are used, that is, there are 11 macro elements in the x direction and 8 macro elements in the y direction. The shell element with five integration points is used to simulate the cell wall. The element type is set to S4R, which is a general shell element type in Abaqus. It has the following properties: a four-node curved shell element that can be used to model thin or thick shell structures, including hourglass mode, and a simplified integration method that allows finite membrane strain. In order to verify the accuracy of the mesh size, 3 mm, 2 mm, 1 mm and 0.8 mm size grids were used for verification. As shown in Fig. 2, the results converge at 1 mm, so the 1 mm grid is used in this paper. In this paper, except for the other stated, the 1 mm grid is used. In the modeling, RHA is placed between two rigid plates, and the out-of-plane displacement is limited. The bottom plate is fixed, and the top plate is squeezed downward at a constant speed. General contact is used for the whole model. The tangential behavior is frictionless, and the normal behavior is 'hard'contact.

**Figure 3:**
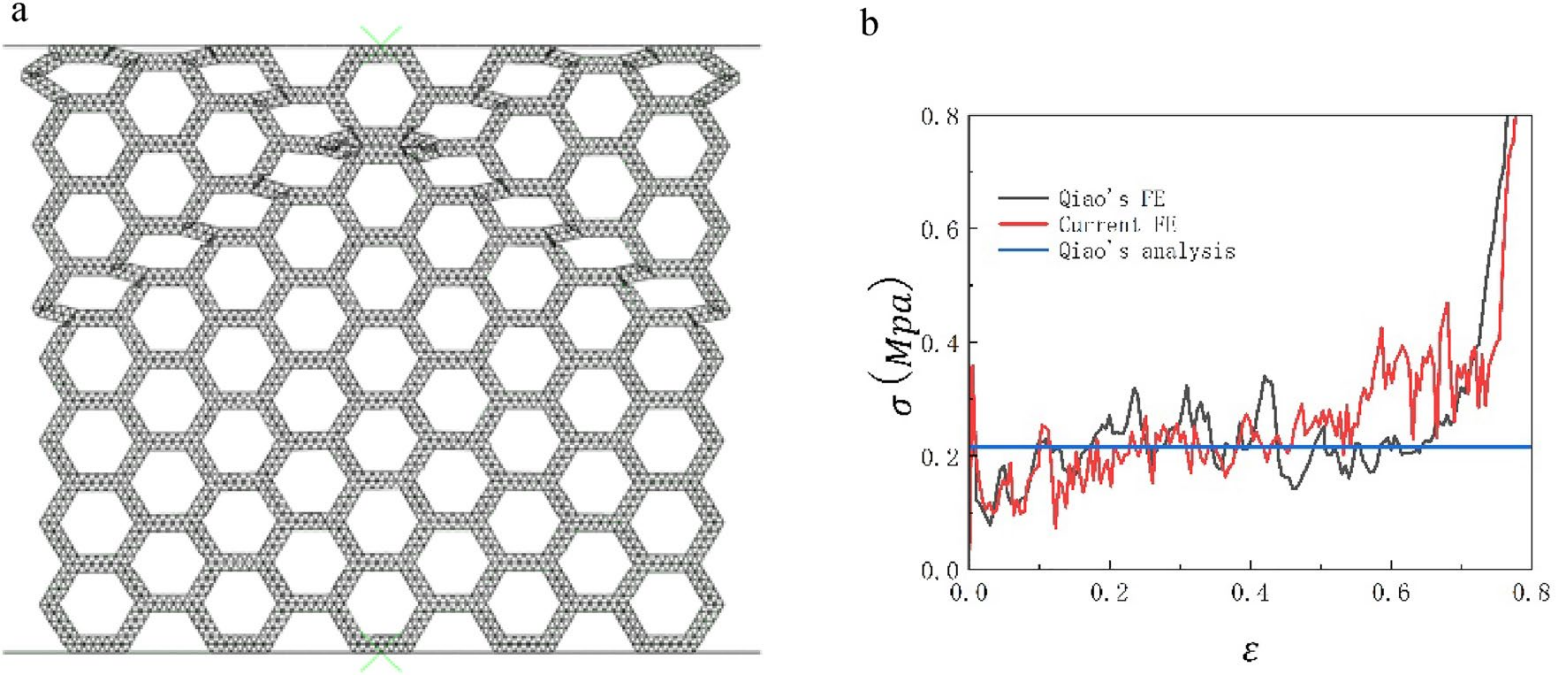
(**a**) SHH deformation mode (**b**) Comparison between finite element simulation and Qiao's analytical solution.

In order to verify the reliability of our finite element model, we simulate and compared the results with Qiao et al.^13^. In the FE simulation, 8 × 9 cells are used, that is, there are 9 macroscopic units in the x direction and 9 macroscopic units in the y direction. The in-plane thickness of the honeycomb plate is b = 2 mm, and the side length of the equilateral triangular substructure is a = 10 mm. The 4-node quadrilateral shell element is adopted, and the density of the shell is set to 0.35 mm andthe relative density is 5%. As shown in Fig. 3, the 'X' deformation mode of SHH obtained by Abaqus software is the same as that obtained by Qiao et al.^13^, and the platform stress obtained by Abaqus software is basically consistent with the simulation results and formula derivation results of Qiao, which can prove that the model in this paper has good accuracy.

## Quasi-static collapse response

### y uniaxial compression

**Figure 4:**
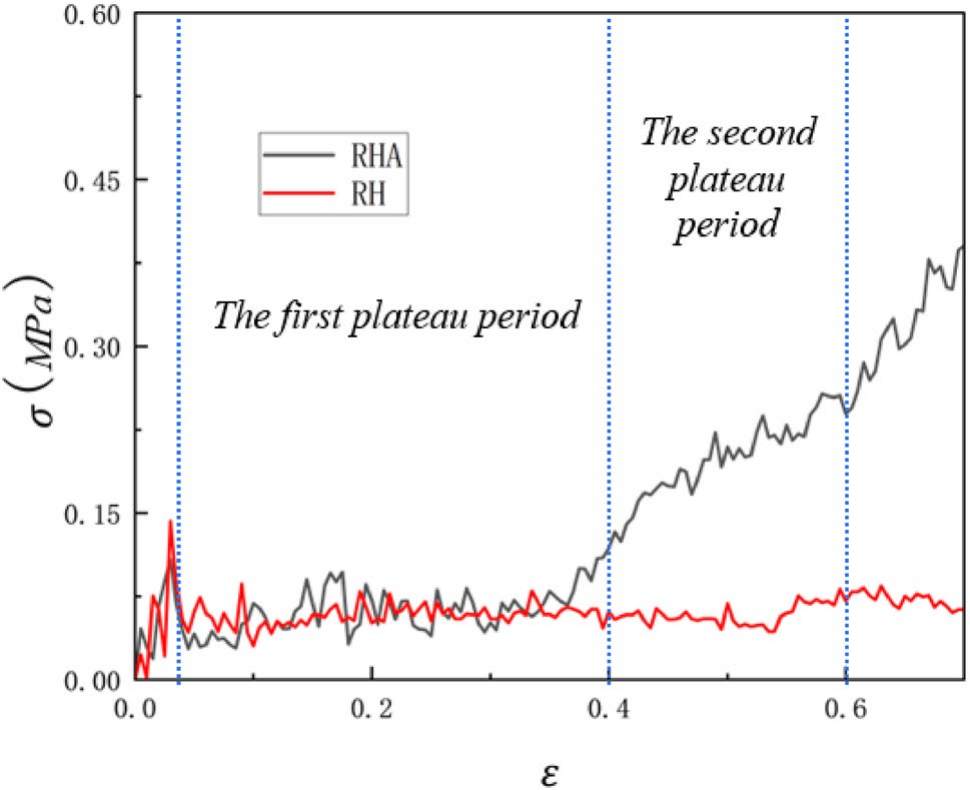
RH RHA simulation comparison.

In this section, the quasi-static collapse of the RHA honeycomb model with N = 7 M = 4 in the y direction under uniaxial compression is analyzed. A constant speed of 1 m/s is applied to the top plate, and the stress–strain curve of the RHA with ρ = 5% is approximately simulated by the finite element simulation. As shown in Fig. 4, it can be seen that the RHA has an obvious shock zone and then enters the platform area. However, unlike the traditional honeycomb structure, the RHA has two plateau periods. Because the tilted edge and the horizontal edge of RHA are different, the RHA has two deformation modes. The first is the normal negative Poisson's ratio re-entrant honeycomb deformation. The deformation mode is compression and torsion of the inclined edge. Thesecond is the further compression of the horizontal edge. This phenomenon will be discussed later.

In order to compare, the stress–strain curves of the first-order reentrant honeycomb (RH) with 8 × 11 non-layered structures (i.e., horizontal and side lengths of 0.07 m and 0.04 m, respectively) under uniaxial compression were simulated by finite element method. It is noted that the cell size used in RH is sufficient to ensure that the simulation value can be converged, and the relative density of RH is consistent with the relative density of RHA, both of which are 5%. It can be seen that the plateau period of RH structure is much longer than that of RHA. When RHA enters the second plateau period, RH has not yet entered the densification stage, which is because the RH structure is too simple and the structure has not been compressed. It can be seen that in the first stage, the platform stress of RH and RHA is basically the same. In the second stage, the platform stress of RHA is increased to 332% of the original, the first platform stress of RHA is 0.06436 MPa, and the second platform stress is 0.2136 MPa. This data is derived from the formula derived later.

#### Collapse mode

**Figure 5:**
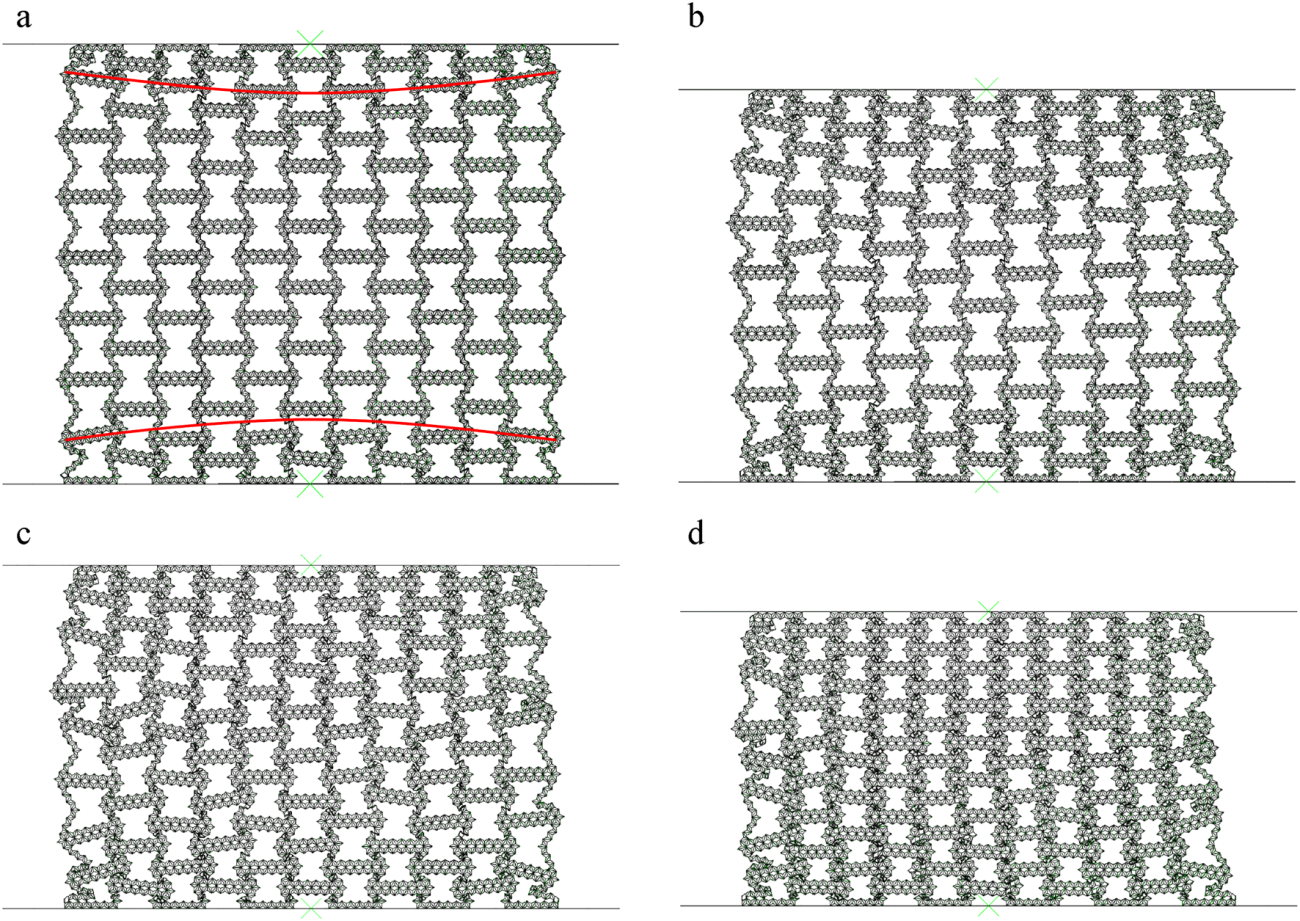
FE predicts RHA deformation for N = 7 M = 4 and $\overline{\rho } = 5\%$ (**a**) $\varepsilon = 10\%$ (**b**) $\varepsilon = 20\%$ (**c**) $\varepsilon = 30\%$ (**d**) $\varepsilon = 40\%$.

**Figure 6:**
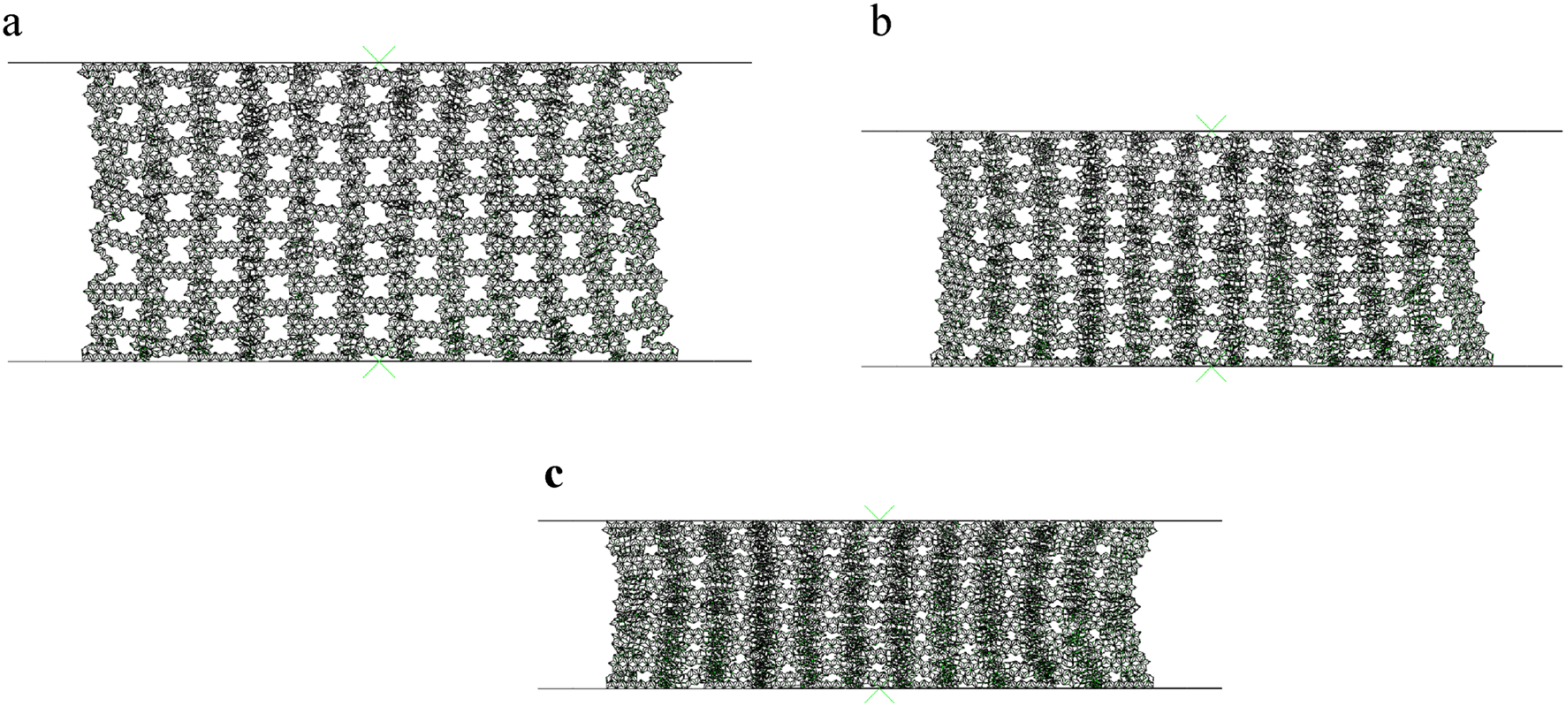
FE predicts the deformation of RHA when N = 7 M = 4 and $\overline{\rho } = 5\%$ (**a**) $\varepsilon = 50\%$ (**b**) $\varepsilon = 60\%$ (**c**) $\varepsilon = 70\%$.

As shown in Fig. 5, it can be seen that even if the deformation is small, the honeycomb has a slight shrinkage in the x direction, which is due to the negative Poisson's ratio structure of the macroscopic cell. Negative Poisson's ratio honeycomb under pressure, due to its special structure, will not expand to both sides, but shrink together. It can be clearly seen from Figs. 5 and 6 that as the compression progresses, the deformed part of the honeycomb shrinks internally. The results obtained from Fig. 5 show that as the compression progresses, the deformation is more and more obvious. The upper and lower parts of the honeycomb are observed to form local bands, which are shown as arc type bands. This is different from the 'v' type bands observed by Tan et al.^15^. Due to the fact that the horizontal edge and the inclined edge of the structure studied in this paper are not of the same size, the transverse contraction is slowed down, resulting in the standard deformation band of the re-enter honeycomb has changed and becoming an arc band.

Upon the global compression reaches at $\varepsilon = 10\%$, the macro cells in the local band in the honeycomb begin to absorb energy through the rotation and compression of the side edges. As the crushing progresses, the arc type band gradually appears at $\varepsilon = 20\%$. At $\varepsilon = 40\%$, all the inclined edge are basically completely compressed, leaving only the horizontal edge edges. At this time, the second stage of compression is carried out. As shown in Fig. 6a, the compression method in the global compression of the honeycomb gradually changes to the compression of the upper and lower bottom edges. At this time, the macrostructure has been compressed, the compression is carried out in the substructure, and the substructure is broken. When the honeycomb is further compressed, the substructure is completely broken, as shown in Fig. 6c, and the material begins to densify.

#### Collapse stress

**Figure 7:**
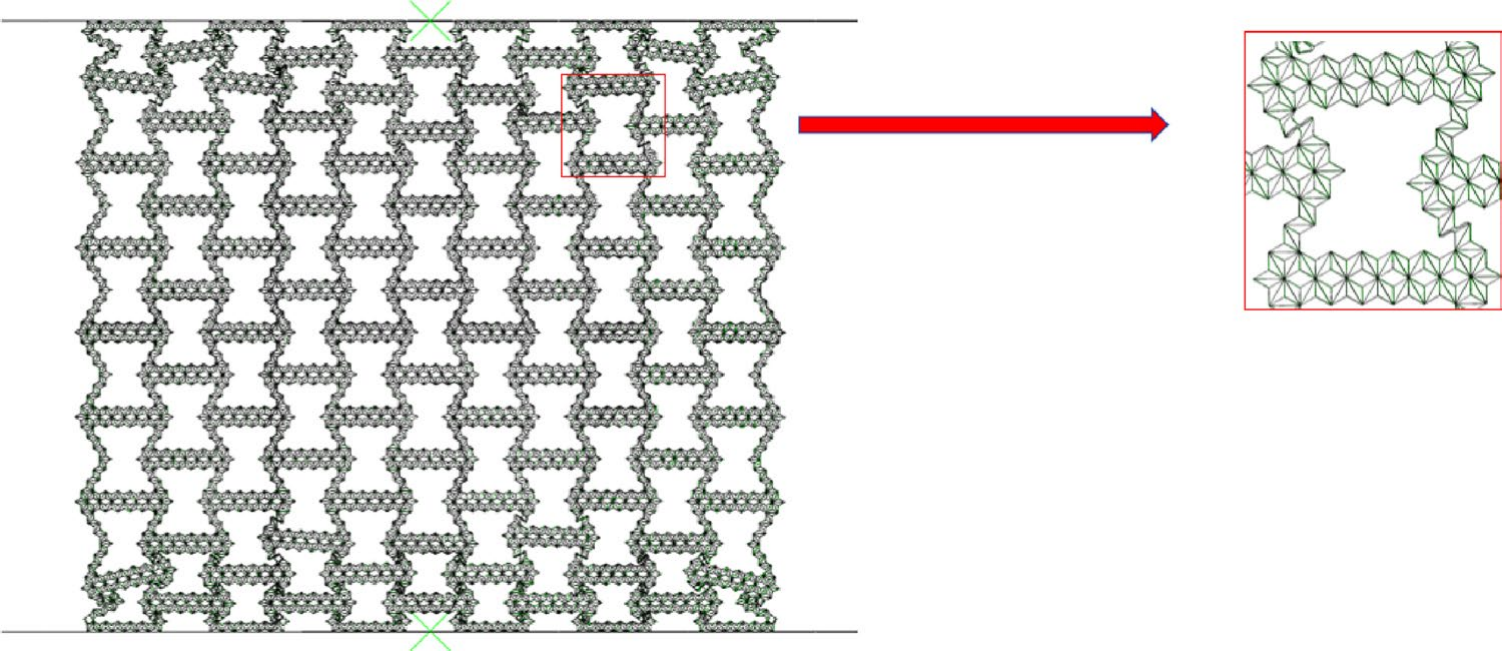
RHA simulation magnification diagram.

**Figure 8:**
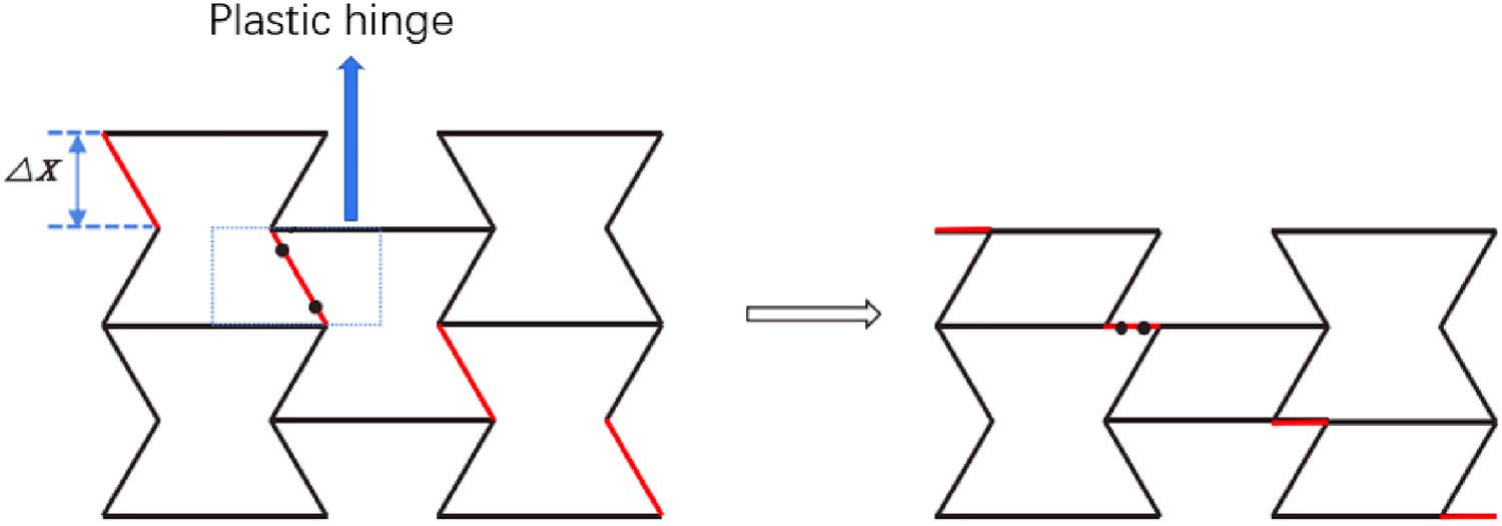
Simplify the enlarged picture.

For the collapse stress of the first stage, we can establish the analytical model of the collapse stress of RHA based on the deformation mode of finite element simulation. In order to facilitate the description of the deformation mode, Fig. 7 is the enlarged figure of the deformation mode of Fig. 5a. The corresponding simplified deformation mode is shown in Fig. 8 In this paper, the two-scale method is used to derive the analysis results. Considering the deformation of the macrostructure, the substructure is transformed into a uniform solid. It can be known from the deformation diagram that the overall failure of the macrostructure is achieved by the collapse of the substructure. Therefore, this method measures the failure of the structure by replacing the initial yield stress of the material with the collapse stress of the sub-result.

As shown in Fig. 7, the initial deformation of the honeycomb is the rotation of the side at the macro level. At this time, the substructure of the horizontal edge and the inclined edge of the honeycomb is basically unchanged. The energy absorption effect of the honeycomb completely depends on the rotation energy absorption effect of the side. The platform stress of the honeycomb is derived from the rotation of the cell wall.

In Fig. 8, only the deformation of the macroscopic cell is considered, where the plastic hinge is marked with a blue wire frame. The deformed red line indicates that the element edges in the deformed region rotate and compress at the same time, which is consistent with the prediction results of finite element simulation. For the RHA deformation process shown in Fig. 7, the displacement of the macro element is $\Delta x = \sqrt 3 (M - 2)a/2$, The force acting on the honeycomb is $F = ab(2N - M)/2\sigma_{y1}$ and the external force work on the element is

2 $$W_{ext} = F \cdot \Delta x = \frac{{\sqrt 3 a^{2} b\left( {2N - M} \right)(M - 2)\sigma_{y1} }}{4}$$

Kindly check and confirm whether the equation (2) is correctly identified.Formula 2 is correct

Here ${\sigma }^{y1}$ is the far-end y-direction stress of the first stage.$W_{ext} = W_{ht} + W_{dt}$.

Then we consider the rotation of the red edge, and the work of the two hinges marked in the blue box is W = 2 ${M}_{P}\Delta \mathrm{\vartheta }$,The cell wall plastic bending moment of the macrostructure is $M_{pt} = 3a^{2} b\sigma_{0} /16$, The rotation angle is $\Delta \mathrm{\vartheta }=\uppi /3$, Therefore, plastic bending dissipation energy is

3 $$W_{ht} = 2M_{pt} \Delta \theta = \frac{{\pi a^{2} b\sigma_{0} }}{8}$$

In addition to considering the plastic dissipation caused by the rotation of the plastic hinge, the energy dissipated by the red edge shortening during compression must also be considered, so the compression dissipation energy is

4 $$W_{dt} = \frac{{\sqrt 3 (M - 2)a^{2} b\sigma_{0} }}{4}$$

Here, ${\sigma }_{0}$ is the collapse stress of the substructure isosceles triangle structure. Through the stress formula obtained by Song et al.^14^, the analytical formula of the substructure stress is

5 $$\sigma_{0} = \frac{{(\pi + \theta_{2} )\sin \theta_{1}^{2} }}{{\sin (\theta_{1} - \theta_{2} )(2\sin \theta_{1} \sin \theta_{2} - \sin \theta_{2} )}}\left( \frac{h}{a} \right)^{2} \sigma$$

**Figure 9:**
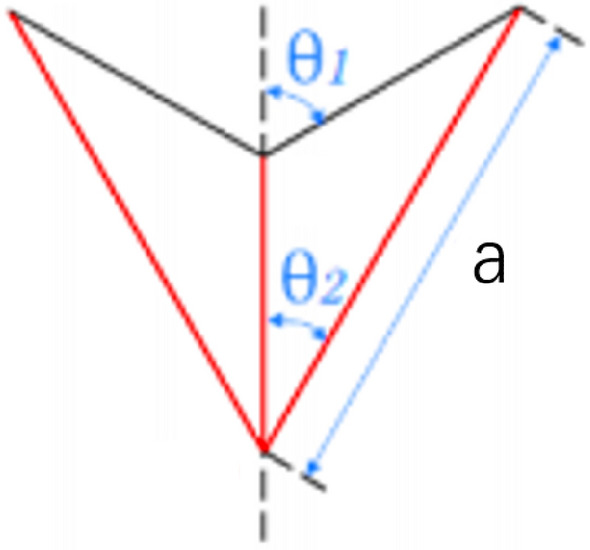
RHA substructure.

$\theta_{1}$ and $\theta_{2}$ are two angles of the triangular word structure respectively. In this paper, $\theta_{1}$ is set to $\uppi$/3, $\theta_{2}$ is set to $\uppi$/6. As shown in Fig. 9

Through the above formula derivation, the Y-direction stress of the first-order compression RHA is

6 $$\sigma_{{y_{1} }} = \frac{{\left[ {\frac{\pi }{8} + \frac{\sqrt 3 }{4}(M - 2)} \right]}}{{\frac{\sqrt 3 }{4}(2N - M)(M - 2)}}\frac{{(\pi + \theta_{2} )\sin \theta_{1}^{2} }}{{\sin (\theta_{1} - \theta_{2} )(2\sin \theta_{1} \sin \theta_{2} - \sin \theta_{2} )}}\left( \frac{h}{a} \right)^{2} \sigma$$

**Figure 10:**
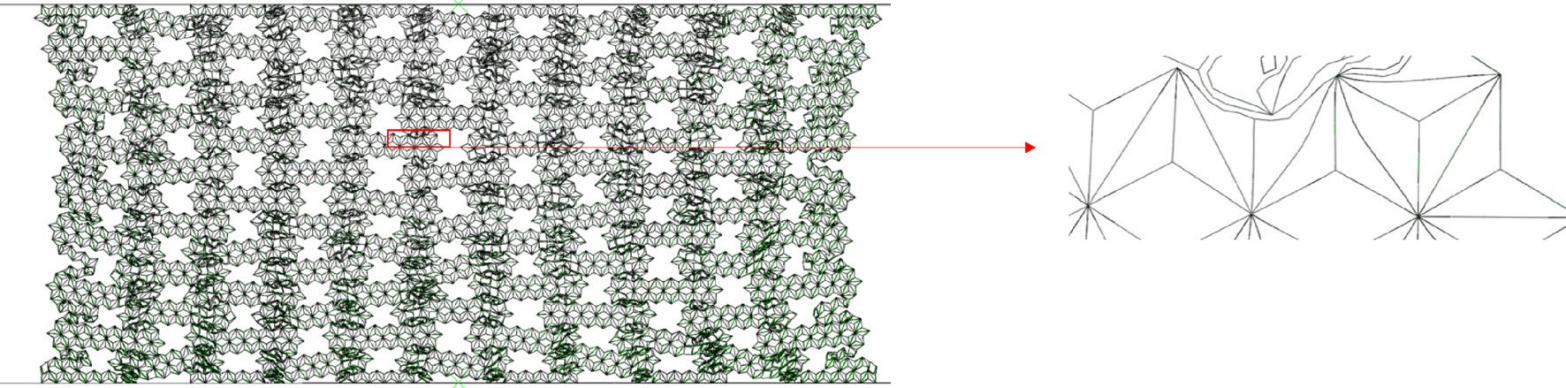
RHA $\varepsilon = 55\%$ deformation mode and enlarged figure.

**Figure 11:**
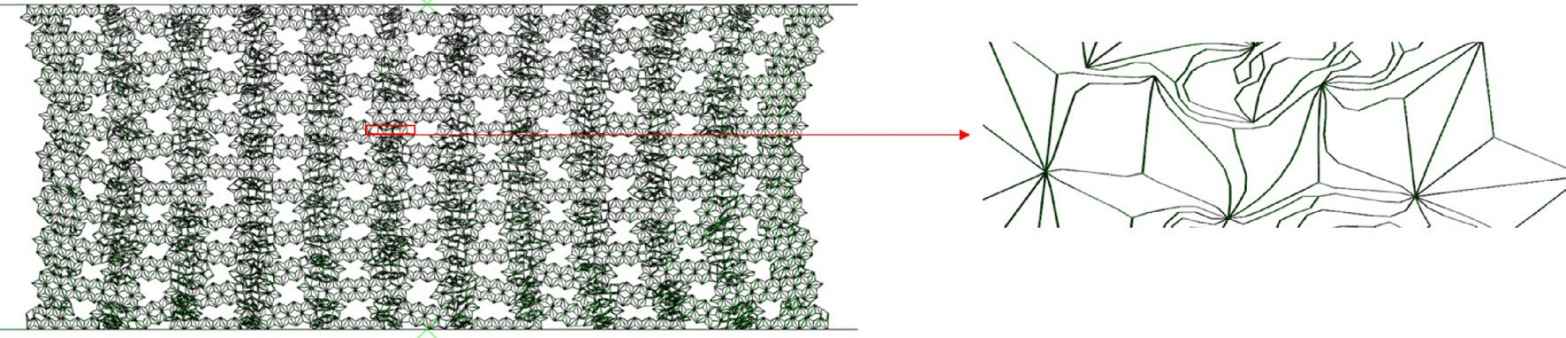
RHA deformation mode and enlarged figure.

**Figure 12:**
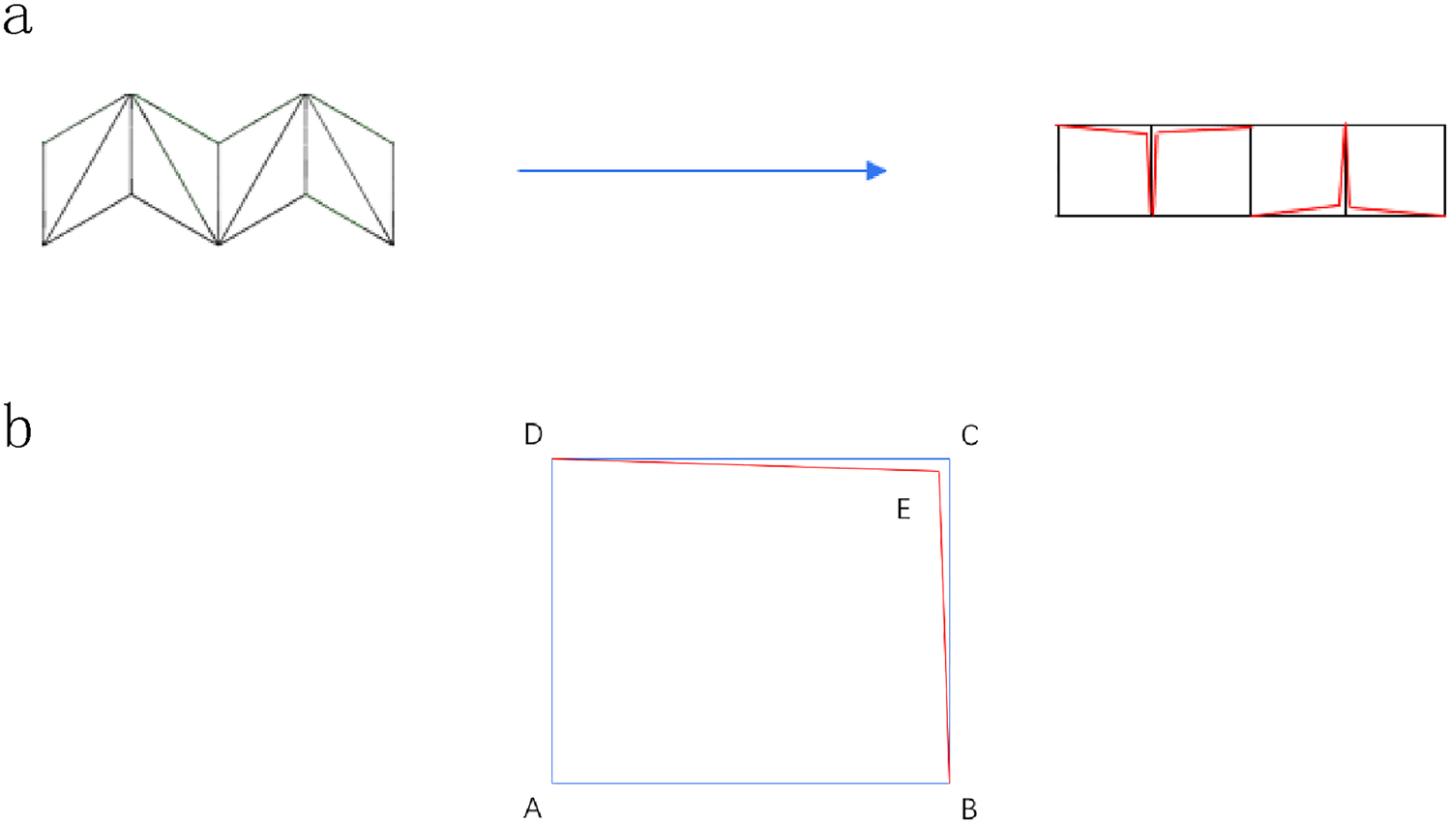
(**a**) Simplified deformation from $\varepsilon = 55\%$ to $\varepsilon = 60\%$ (**b**) Single substructure amplification diagram.

For the second-stage collapse stress, we established an analytical model of the second-stage collapse stress through the collapse model of finite element simulation. Similarly, for the sake of illustration, Figs. 10 and 11 are enlarged images of the deformation mode during the second-order compression. This stage corresponds to the second plateau period of the honeycomb structure. It can be seen from Figs. 10 and 11 that the honeycomb side is compressed and compacted at this stage, and the subsequent deformation is only the compression of the bottom side. At this time, the platform stress completely depends on the deformation of the bottom side substructure, and the energy absorption effect is related to the substructure of the honeycomb bottom side. In order to facilitate the understanding of the deformation mode, the microstructure is structured into a simplified image shown in Fig. 12a. We can see that when the compression of the horizontal edge begins at $\varepsilon = 40\%$, the actual compression mode is similar to the compression of the horizontal edge laminated together. At this time, we choose the process of deformation from $\varepsilon = 55\%$ to $\varepsilon = 60\%$ derive the collapse stress analytical model of the second stage of deformation.

It can be seen from Fig. 12 that the substructure changes from the overall diamond structure to the approximate square structure during compression, and the main deformation is the plastic rotation around the node. The work of an external force exerted on a cell with displacement $\Delta x = a/2\sqrt 3$ is

7 $$W_{ext} = \frac{{5a^{2} b\sigma_{y2} }}{2\sqrt 3 }$$

Here ${\sigma }_{y2}$ is the distal stress of the second stage.

We divide the deformation diagram of Fig. 12a into four parts. From Fig. 12b, it can be seen that each part can be divided into five hinges when pressed. The rotation angle of such a part around the hinge is 5 $\uppi$/3, so the total rotation angle is 20 $\uppi$/3.

The plastic dissipation of the rotating edge is

8 $$W_{ht} = 2M_{pt} \Delta \theta = \frac{{10\pi h^{2} b\sigma }}{3}$$

The second stage y to the distal stress

9 $$\sigma_{y2} = \frac{4\pi }{{\sqrt 3 }}\left( \frac{h}{a} \right)^{2} \sigma$$

**Figure 13:**
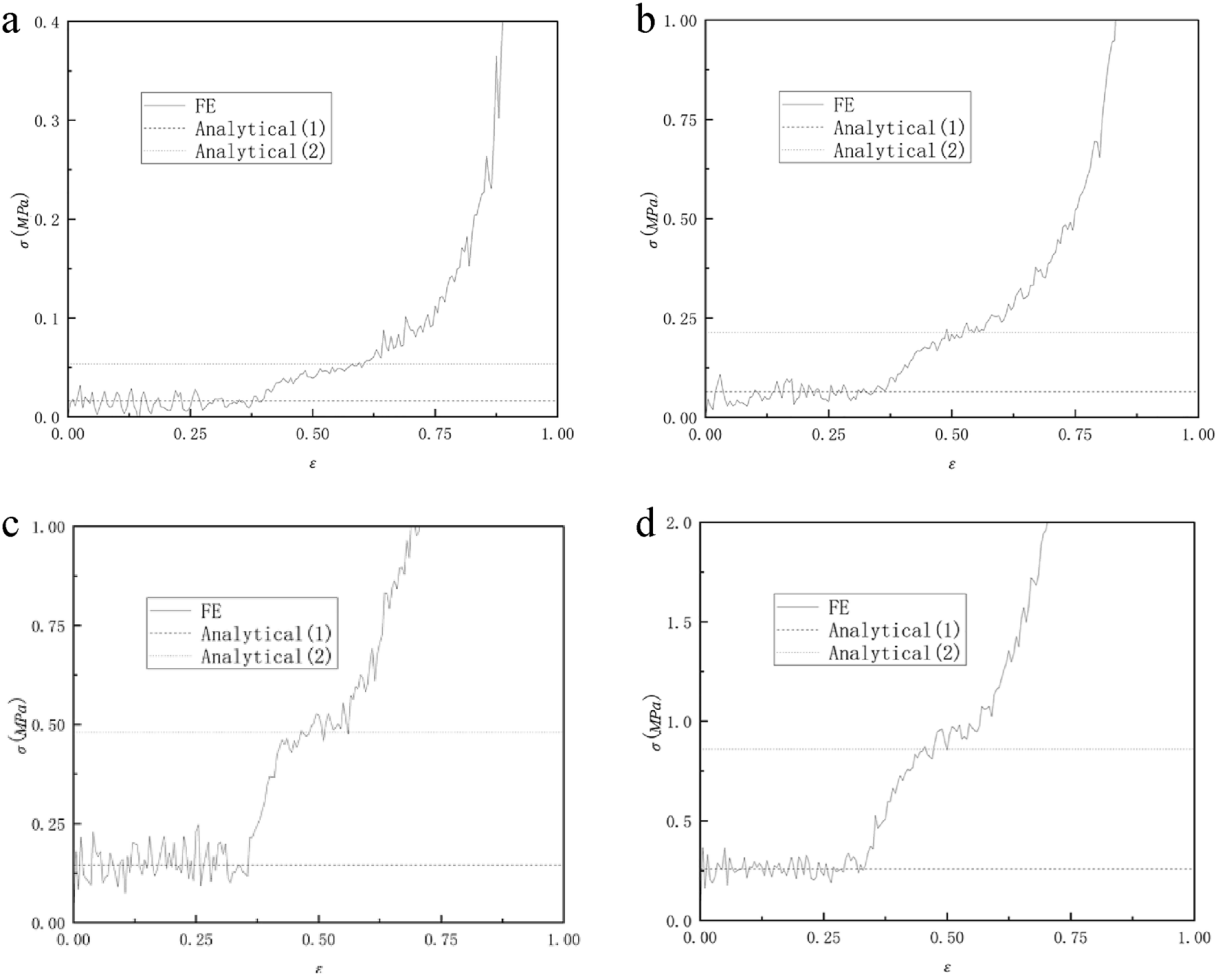
Relative density (**a**) 2.5%, (**b**) 5%, (**c**) 7.5% and (**d**) 10% RHA analytically predicted collapse stress and finite element predicted stress–strain response.

Figure 13 shows the comparison between the analytically predicted collapse stress and the finite element predicted stress–strain response of RHA when the relative density is 2.5%, 5%, 7.5%, and 10%. We can see from Fig. 13 that the analytical predicted collapse stress in the first stage is in good agreement with the finite element predicted results. The analytically predicted collapse stress in the second stage has some deviations from the finite element predicted results, but the deviations are within the controllable range. This is due to the fact that the structural connection of RHA after compression is not very close, resulting in the transmission of force is not very continuous. Therefore, the collapse stress of the second stage of analytical prediction can only be roughly the same as that of finite element prediction, and the error is within the controllable range. It can be seen from the formula 6 and the formula that the platform stress of the honeycomb increases with the increase of the thickness of the honeycomb wall under the condition that other conditions remain unchanged. In the case of other parameters unchanged, the thickness of the cell wall of the honeycomb is related to the relative density, so as the relative density of the honeycomb increases, the stress of the honeycomb platform increases, and this trend can also be seen well through Fig. 13.

### x uniaxial compression

**Figure 14:**
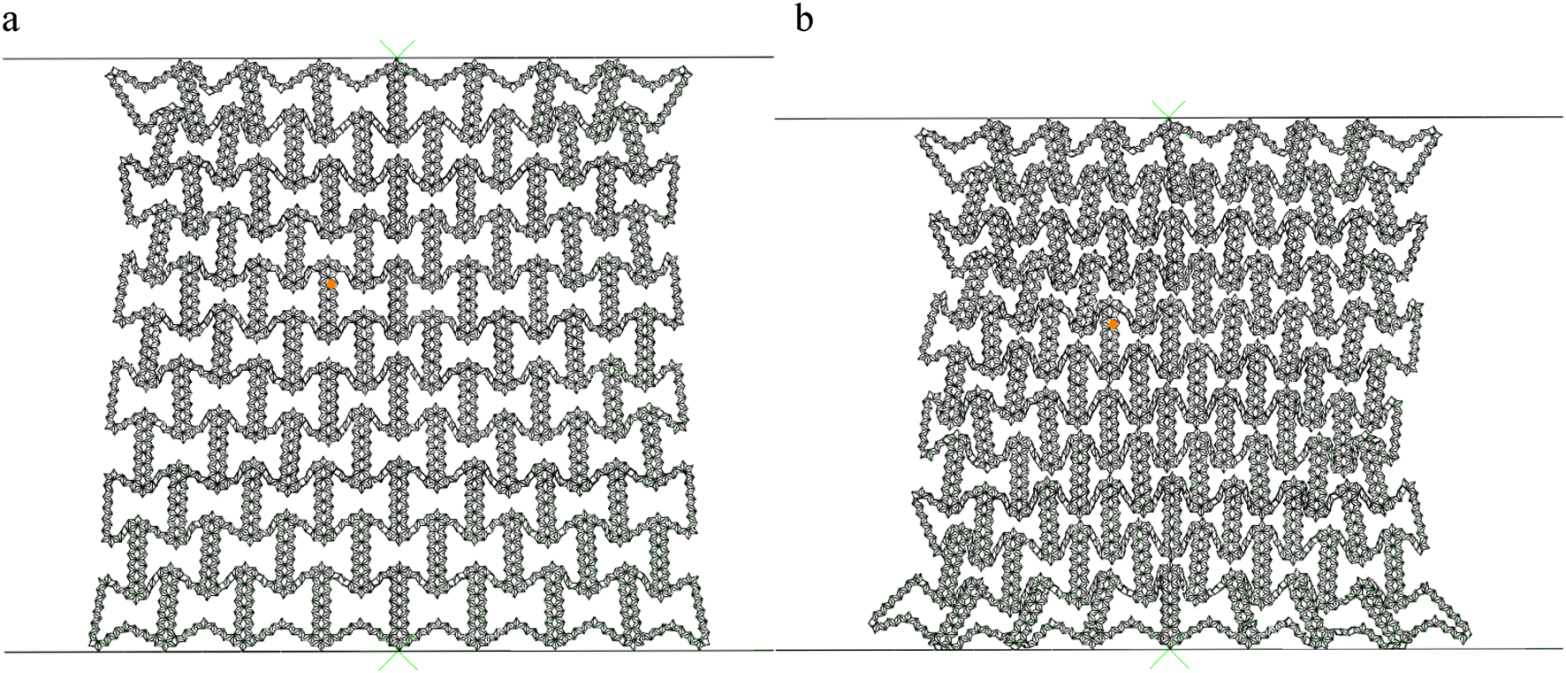
FE predicts the deformation of RHA when N = 7 M = 4 and. $\overline{\rho } = 5\%$ (**a**) $\varepsilon = 10\%$ (**b**) $\varepsilon = 20\%$.

In the previous section, we analyzed the collapse process of RHA under quasi-static compression in the y direction. In this section, we study the quasi-static crushing of RHA under uniaxial compression in the x direction. Figure 14 shows the N = 7 M = 4 and the first stage deformation process of RHA under uniaxial compression. Different from the arc crushing in the y direction, the crushing of RHA in the x direction collapses from top to bottom. Through Fig. 14, we can clearly see the deformation process of honeycomb as a negative Poisson's ratio. As the honeycomb is compressed, through the rotation deformation of the honeycomb wall, the two sides of the honeycomb shrink to the middle, and finally gradually gather together and densify.

#### Collapse mode

**Figure 15:**
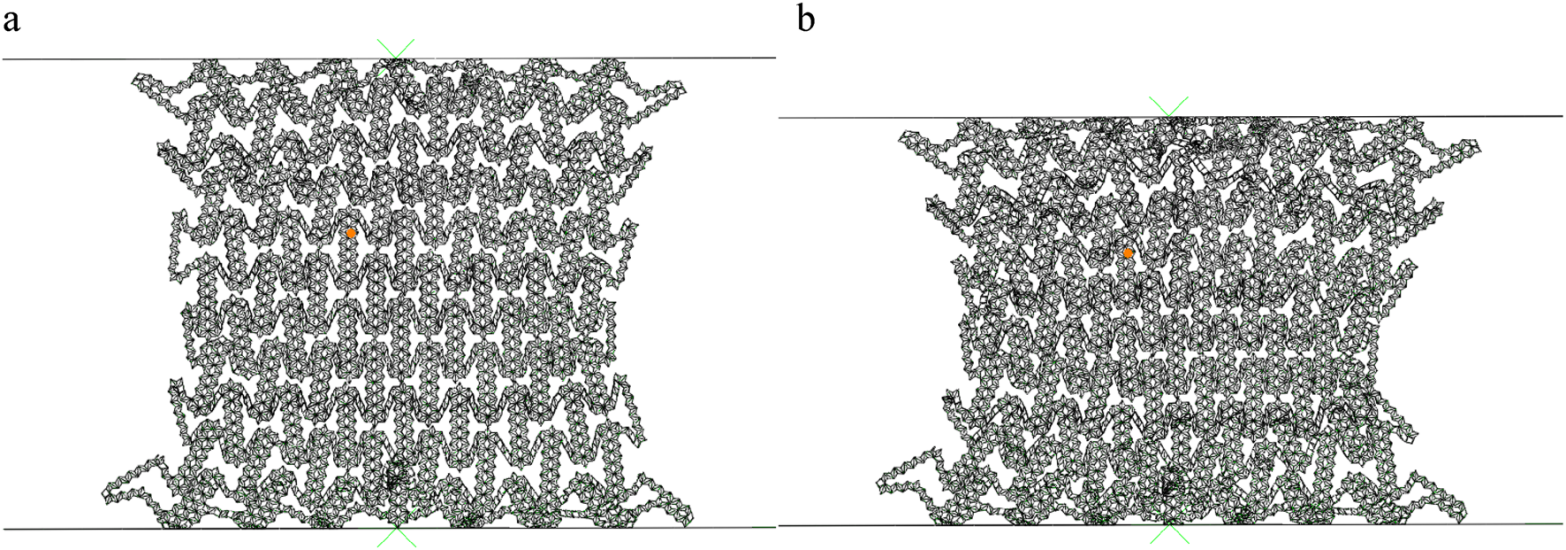
FE predicts the deformation of RHA when N = 7 M = 4 and. $\overline{\rho } = 5\%$ (**a**) $\varepsilon = 30\%$ (**b**) $\varepsilon = 40\%$.

Through Fig. 14, it can be seen that when the RHA deformation is $\varepsilon = 10\%$ to $\varepsilon = 20\%$, the plastic rotation occurs on the side. It can be seen that the collapse is transmitted downward from the contact surface. It can be seen that the honeycomb deformation at this time is similar to the first stage of y-direction compression, both of which are side rotations. Unlike y-direction deformation, the first stage of x-direction compression is two side rotations. At this time, the platform stress of the honeycomb depends on the energy absorption capacity of the side rotation. When the strain is $\varepsilon = 20\%$, the horizontal edge of the macrostructure are basically contracted together. At this time, the structure undergoes second-order deformation as shown in Fig. 15. The second-order deformation is the uniaxial compression of the horizontal edge.

#### Collapse stress

**Figure 16:**
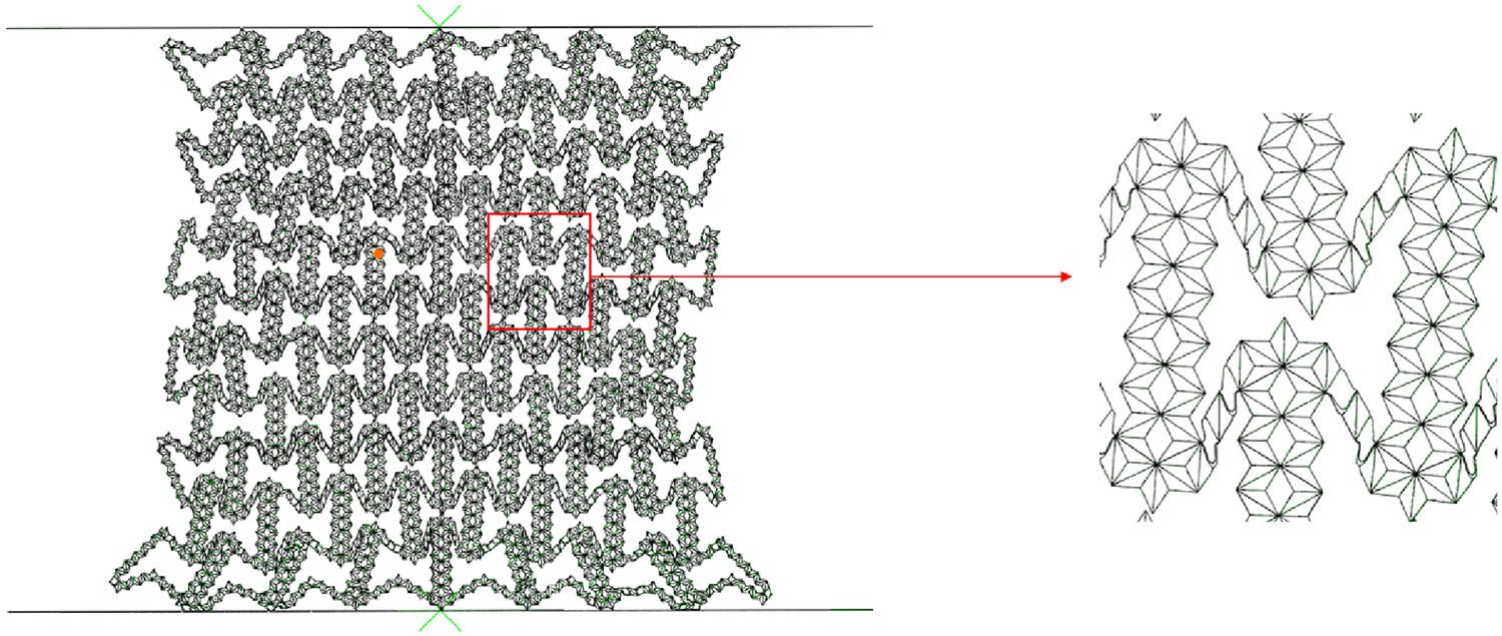
Fig. 14b enlarged figure.

**Figure 17:**
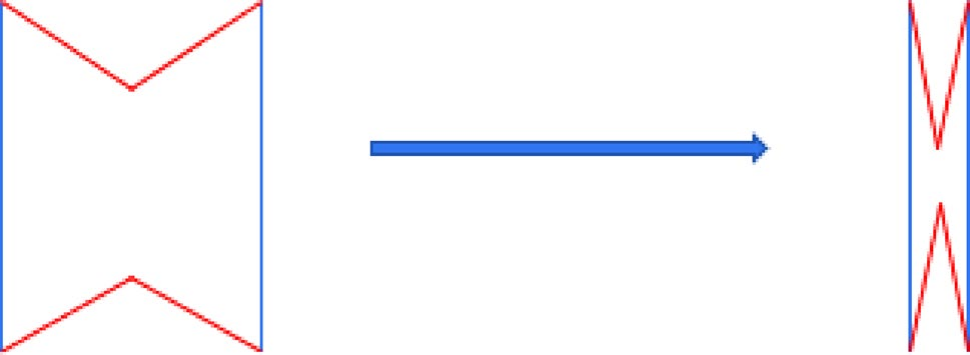
x to the first order deformation simplified figure.

Figure 16 is the structure magnification diagram at $\varepsilon = 20\%$, and Fig. 17 is the simplified model at the first-order deformation. It can be seen that the red edge of Fig. 17 rotates to be completely attached to the horizontal edge when the deformation ends.

The work of an external force exerted on a cell with displacement $\Delta x = Ma/2$ is

10 $$W_{ext} = \frac{{\sqrt 3 M^{2} a^{2} b\sigma_{x} }}{4}$$

Here ${\sigma }_{x1}$ is the first x-direction stress at the far end.

Plastic bending moment $M_{pt} = 3a^{2} b\sigma_{0} /16$ of red edge rotation, rotation angle $\Delta \mathrm{\vartheta }=\uppi /3$, so plastic bending dissipation energy is

11 $$W_{ht} = 2M\Delta \theta = \frac{{ba^{2} \pi \sigma_{0} }}{4}$$

The first x-direction stress

12 $$\sigma_{x} = \frac{\pi }{{\sqrt 3 M^{2} }}\frac{{(\pi + \theta_{2} )\sin \theta_{1}^{2} }}{{\sin (\theta_{1} - \theta_{2} )(2\sin \theta_{1} \sin \theta_{2} - \sin \theta_{2} )}}\left( \frac{h}{l} \right)^{2} \sigma$$

**Figure 18:**
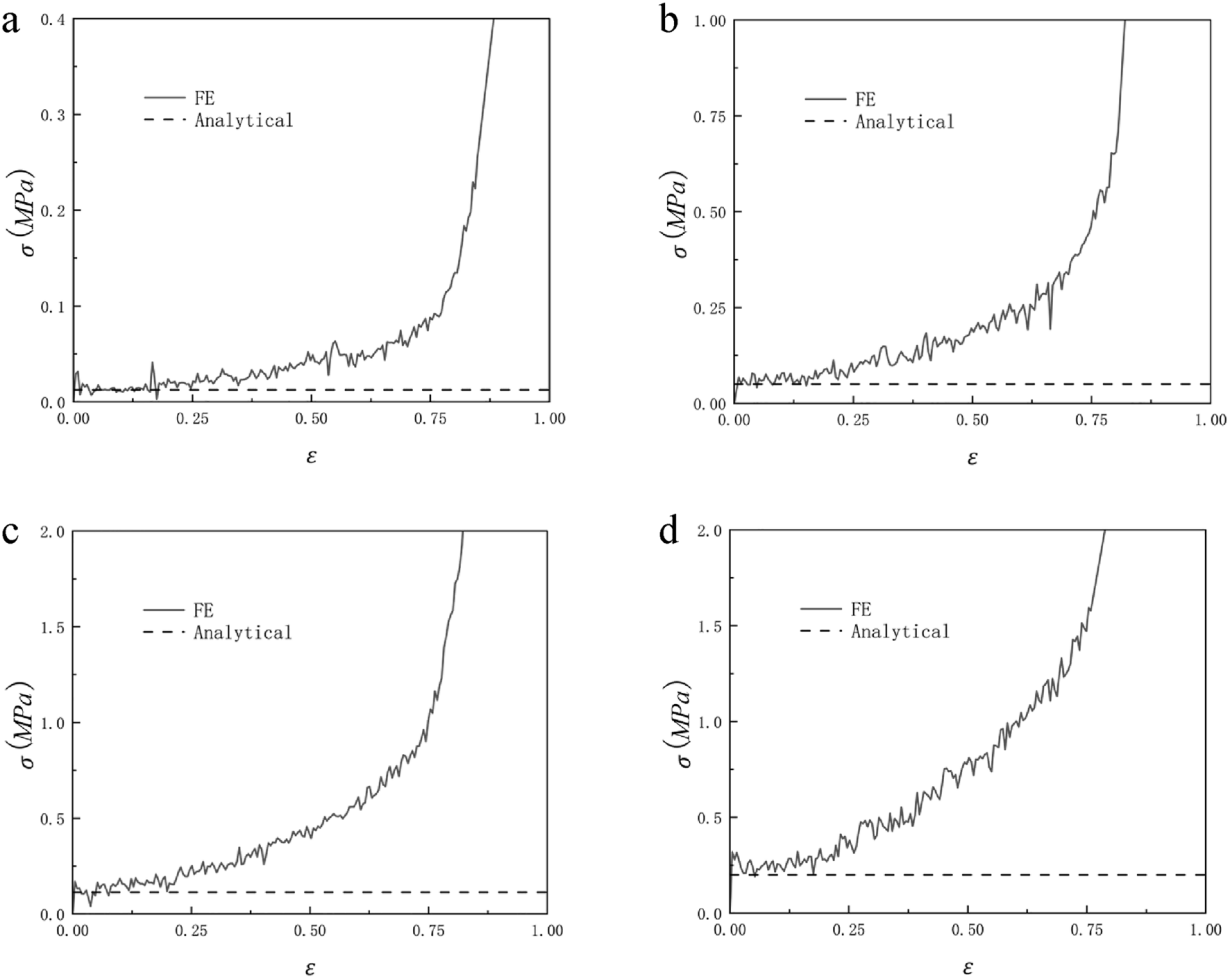
Analytical prediction of collapse stress and finite element prediction of stress–strain response of relative density (**a**) 2.5%, (**b**) 5%, (**c**) 7.5% and (**d**) 10% RHA.

Figure 18 shows the comparison between the analytical failure stress of RHA with a relative density of 2.5%, 5%, 7.5%, and 10% in the x direction and the finite element predicted stress–strain curve. It can be seen from the figure that before $\varepsilon = 20\%$, the analytical stress is in good agreement with the finite element. However, as the compression progresses, the stress of the finite element simulation gradually loses its regularity. It can be seen from Fig. 16 that this is because there are still many gaps in the RHA honeycomb after the first-order deformation, and these gaps gradually disappear with the compression. Therefore, there is no platform period at this stage, and the collapse stress cannot be inferred. It can be seen from Formula 12 that, similar to the trend of quasi-static impact in y direction, the plateau stress of honeycomb structure increases with the increase of relative density, which is consistent with the results shown in Fig. 18.

## Dynamic collapse response

**Figure 19:**
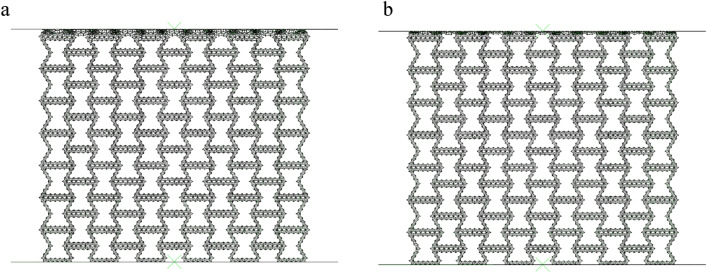
Finite element prediction $\varepsilon = 20\%$ $\overline{\rho } = 5\%$ (**a**) 50 m/s (**b**) 150 m/s.

In this section, we analyze the dynamic collapse response of RHA and study the impact response from 10 to 100 m/s in the y and x directions. Figure 19 shows the dynamic impact response of 50 m/s and 100 m/s. Different from the quasi-static response, the progressive collapse characteristics are presented in the dynamic impact, and the layer-by-layer characteristics are more obvious at 150 m/s.

According to the deformation mode, the impulse of the external force can be obtained by the momentum conservation theory.

13 $$I = A\int_{0}^{t} {(\sigma_{d} - \sigma_{q} )dt}$$

Here ${\sigma }_{d}$ is the dynamic collapse stress, A is the cross-sectional area of the cell t=${\varepsilon }_{d}$ H/V is the duration of the cell from the beginning of collapse to densification, H is the effective height of the cell, ${\varepsilon }_{d}$ is the densification strain of the structure. In the study of Qiao et al.^13^, when the speed reaches 200 m/s, it is close to the ideal degree of densification 1 − $\overline{\rho }$. However, through finite element simulation, in RHA, we can see that when the speed reaches 150 m/s, the densification is close to the ideal, so we can get the strain expression of densification.

14 $$\varepsilon_{d} = \left\{ {\begin{array}{*{20}c} {(0.8 + 0.2)V/V_{0} } & {{\text{V}} \le {\text{V}}_{0} } \\ {(1 - \overline{\rho })} & {{\text{V > V}}_{0} } \\ \end{array} } \right.$$

Here ${\text{V}}_{0}$ is determined by the microstructure and sample size, in this paper ${\text{V}}_{0}$ = 150 m/s.

The momentum change of the structure can be expressed as

15 $$\Delta P = AH\overline{\rho }\rho V$$

Here H is the height of a single re-entrant honeycomb cell. Through the law of conservation of momentum can be obtained

16 $$I = \Delta P$$

Therefore, the dynamic collapse stress expression can be obtained as

17 $$\sigma_{d} = \sigma_{q} + \rho \overline{\rho }\frac{{V^{2} }}{{\varepsilon_{d} }}$$

Here $\sigma_{q}$ is quasi-static stress.

**Figure 20:**
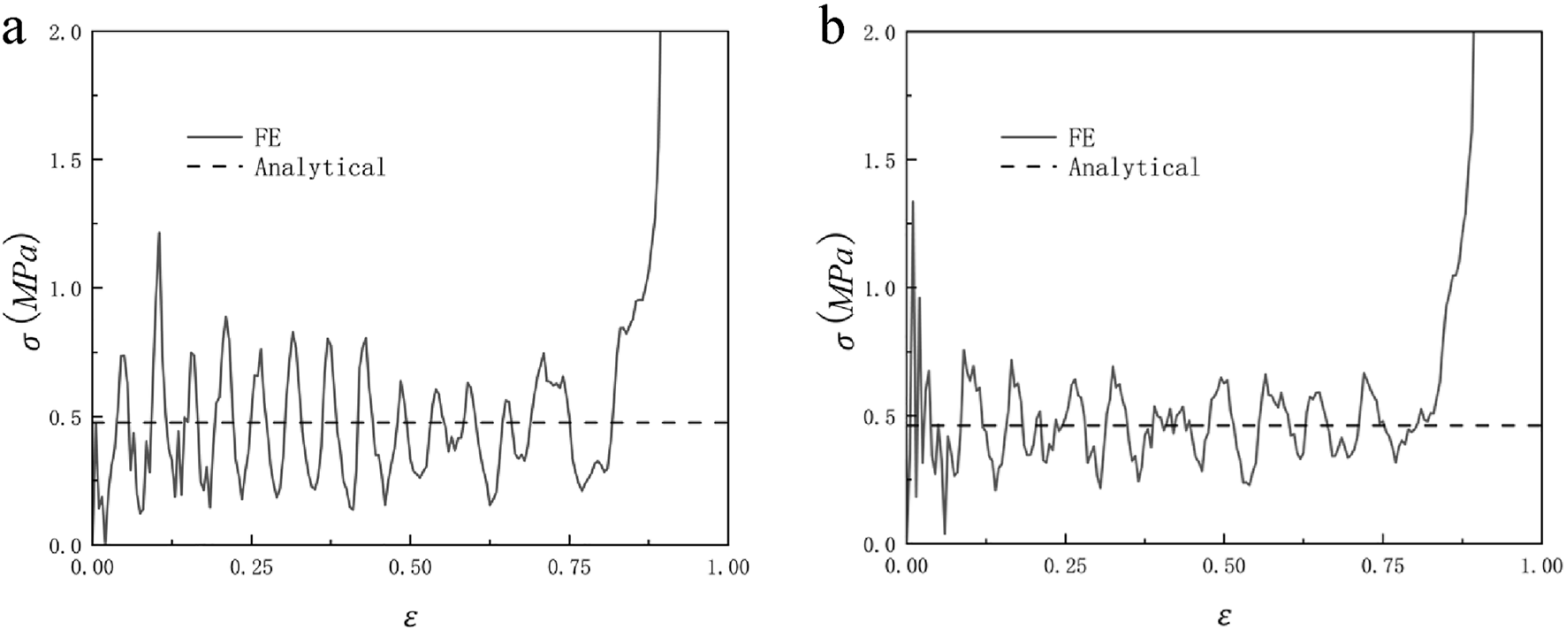
N = 7, M = 4, $\overline{\rho } = 5\%$, V = 50 m/s (**a**) y to impact (**b**) x to impact.

Equation (17) shows that the plateau stress of honeycomb under impact is related to quasi-static stress, density and impact velocity. When the relative density of the honeycomb is constant, the wall thickness of the honeycomb will not change, so the platform stress of the honeycomb will not change. Therefore, the platform stress of the honeycomb under impact depends on the impact velocity (the impact velocity does not exceed 150 m/s). The platform stress of the honeycomb also increases with the increase of the impact velocity, which is consistent with the subsequent simulation results. Figure 20 shows the dynamic stress–strain curve of RHA when N = 7, M = 4, $\overline{\rho } = 5\%$, and impact velocity V = 50 m/s. For the loading in y and x directions, the theoretically predicted collapse stress is in good agreement with the stress–strain response predicted by the finite element model.

**Figure 21:**
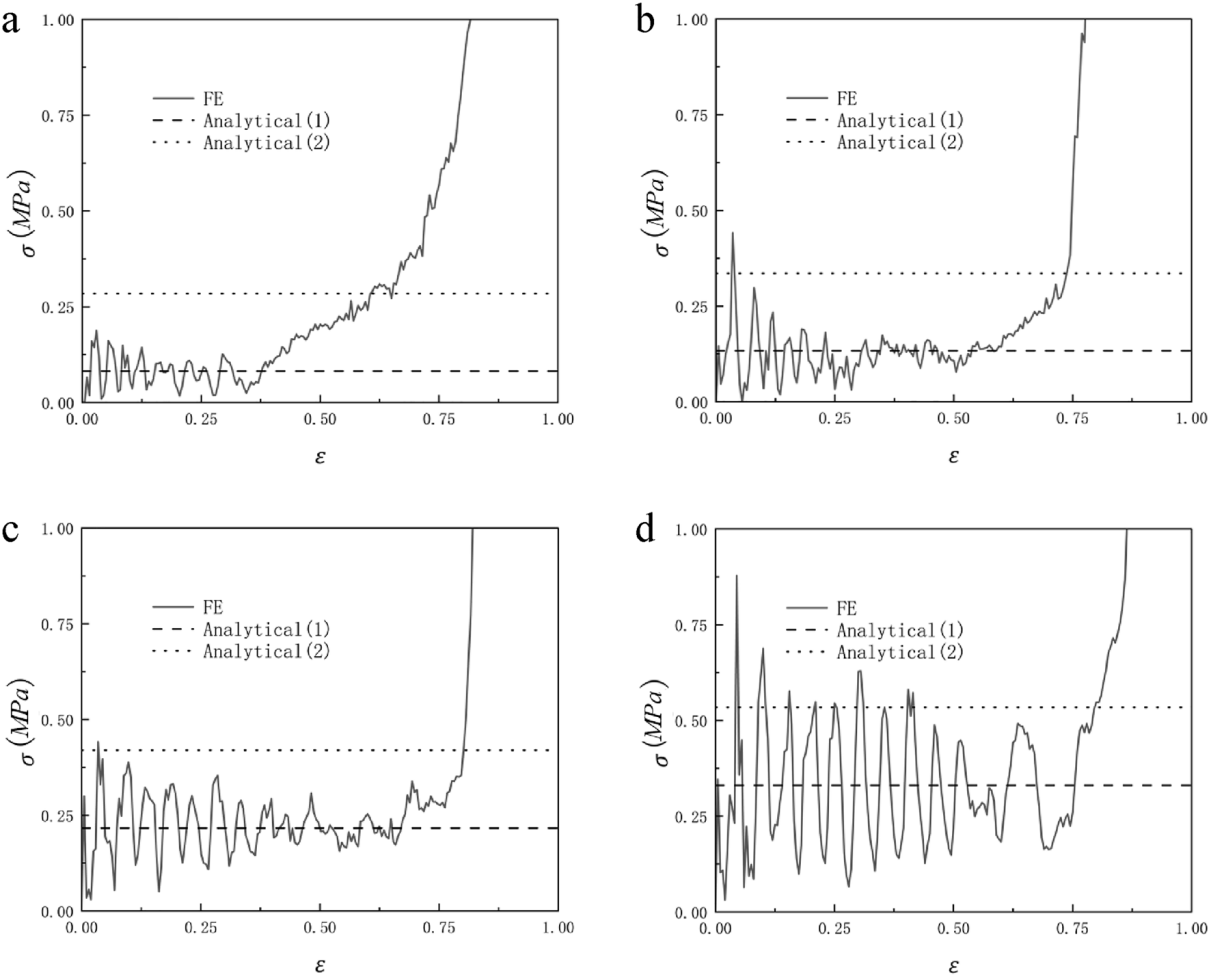
N = 7, M = 4, $$\overline{\rho } = 5\%$$, y direction impact (**a**) 10 m/s (**b**) 20 m/s (**c**) 30 m/s (**d**) 40 m/s.

**Figure 22:**
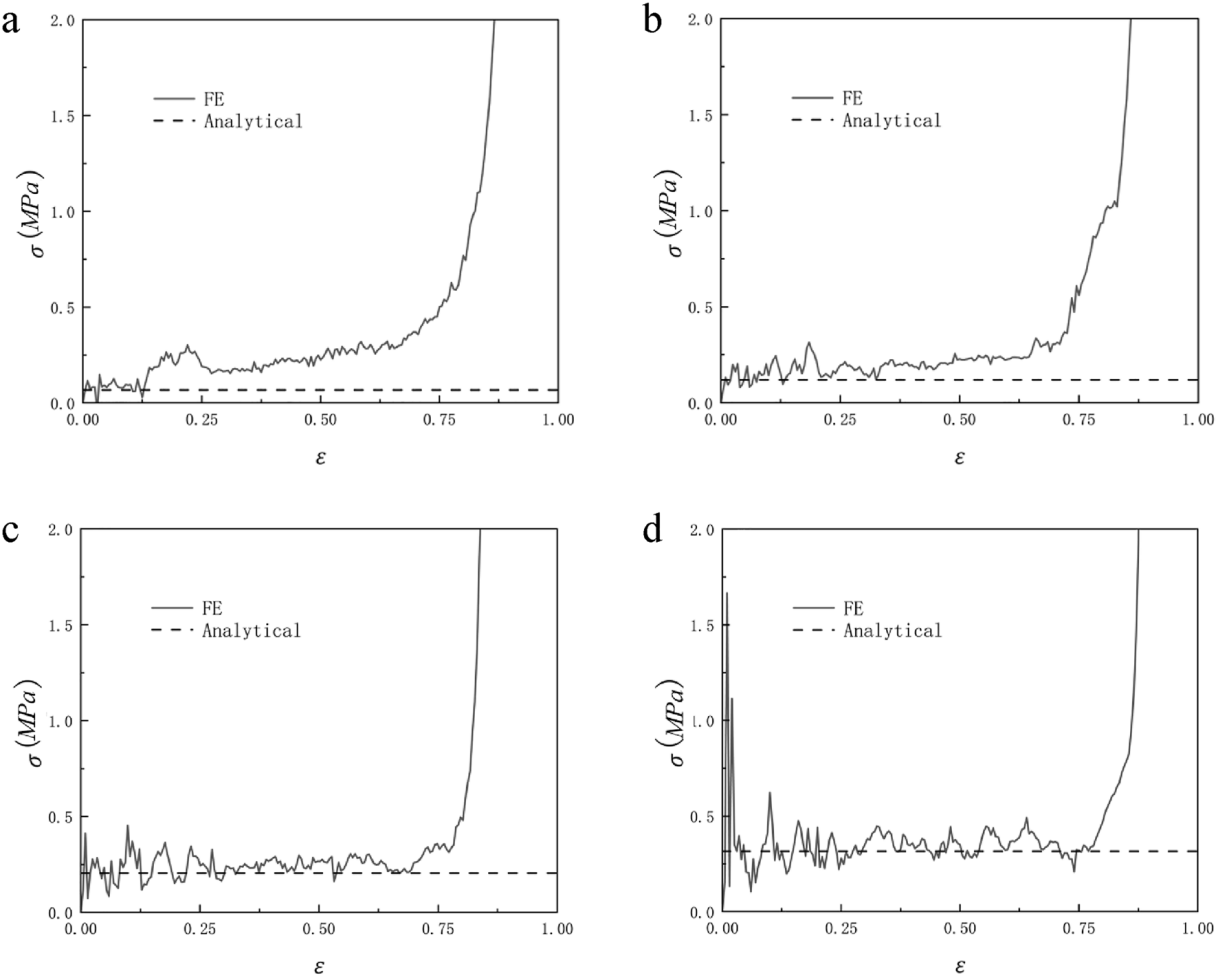
N = 7, M = 4, $$\overline{\rho } = 5\%$$, x direction impact (**a**) 10 m/s (**b**) 20 m/s (**c**) 30 m/s (**d**) 40 m/s.

**Figure 23:**
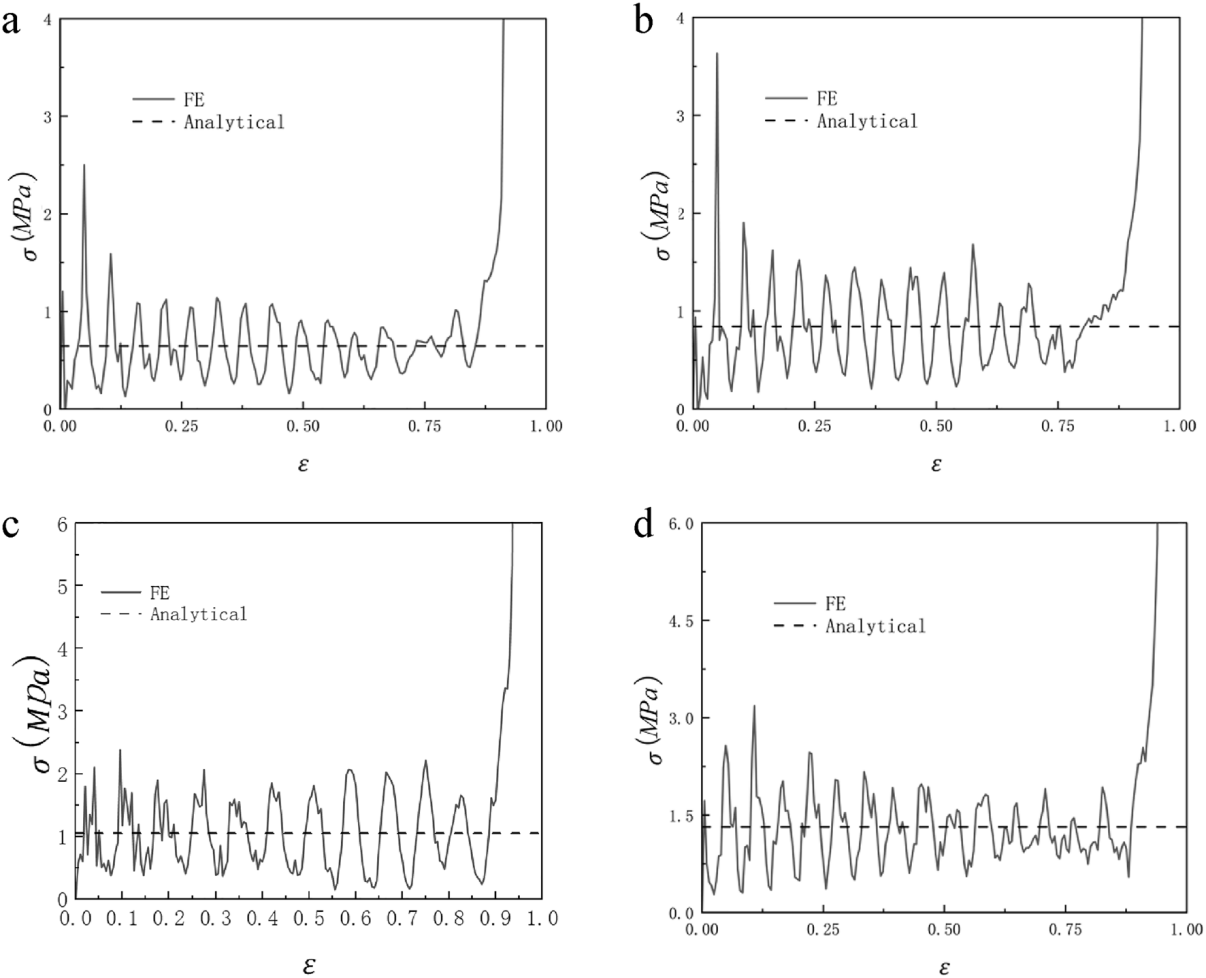
N = 7, M = 4, $$\overline{\rho } = 5\%$$, y direction impact (**a**) 60 m/s (**b**) 70 m/s (**c**) 80 m/s (**d**) 90 m/s.

**Figure 24:**
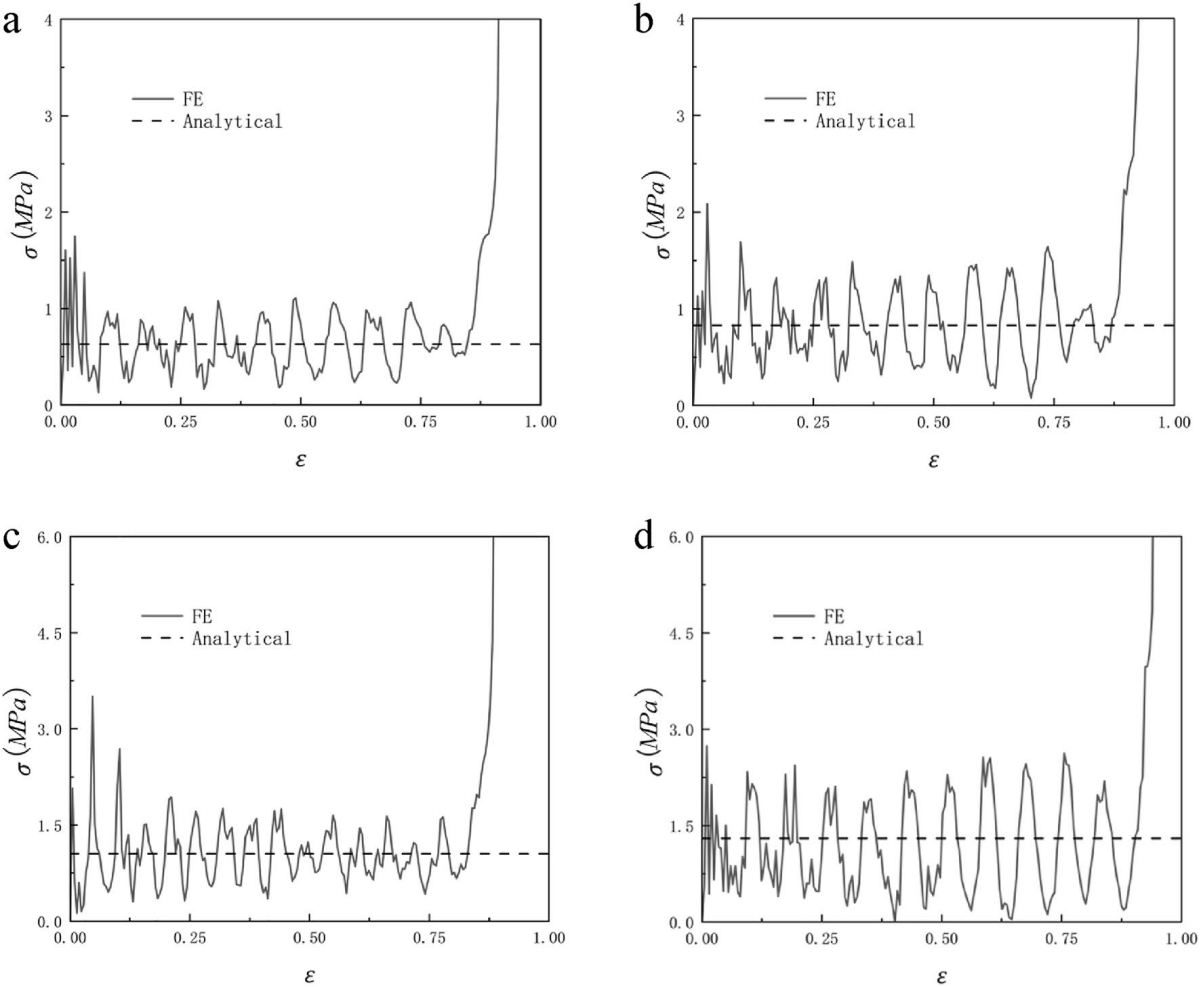
N = 7, M = 4, $$\overline{\rho } = 5\%$$, x direction n impact (**a**) 60 m/s (**b**) 70 m/s (**c**) 80 m/s (**d**) 90 m/s.

When N = 7 and M = 4, the honeycomb is impacted in the y and x directions and the impact velocity is from 10 to 40 m/s, the RHA dynamic impact response is shown in Figs. 21 and 22. We can see that the stress–strain curve obtained by finite element simulation also has two plateau periods when the impact is in the y direction. With the increase in speed, the second plateau period gradually disappears. This is because when the speed is low, the substructure of RHA will not be destroyed for the first time and will experience deformation like static impact. The second stage is equivalent to the superposition of dynamic impact the horizontal edge. With the increase in speed, this phenomenon gradually disappears because the substructure is destroyed. The stress of the second plateau period can be calculated as long as ${\sigma }_{y0}$ is replaced by the second-order quasi-static stress. The same is true in the x direction, but in the x direction, the second platform stress cannot be calculated due to the irregular deformation of the structure. Through numerical simulation and finite element analysis, it can be seen that as the speed continues to increase, the first platform stress and the second platform stress of the honeycomb model under impact also increase. The increase of the speed also causes the fluctuation of the platform stress to become more obvious, which is due to the more severe deformation of the honeycomb. Under the impact of velocity of 10 m/s and 20 m/s, the dynamic impact stress–strain trend in the x direction is similar to that under quasi-static impact. The honeycomb ends the plateau period and enters the densification stage when the deformation is very small. As the speed reaches 30 m/s, this phenomenon begins to disappear. When the speed reaches 40 m/s, this appearance disappears completely. Figures 23 and 24 are the dynamic response impact and predicted stress comparison of N = 7, M = 4, $\overline{\rho } = 5\%$, and the impact speed from 60 to 90 m/s. Under high-speed impact, we can see that the second plateau period disappears with the increase in speed. It can be seen from Figs. 23 and 24 that the stress analytical solution and the stress–strain curve obtained by finite element software simulation are well-fitted.

## Concluding remarks

In this paper, the in-plane crashworthiness of a new type of re-entrant hierarchical honeycomb structure under dynamic and static impact is studied by means of numerical analysis and finite element simulation. The energy absorption capacity of honeycomb under different relative densities and impact velocities are discussed respectively. Previous studies on the honeycomb have noted the second platform stress of the honeycomb, but they have not carried out detailed calculations. This paper innovatively calculates the second platform stress of the honeycomb. RHA honeycomb also clearly reflects the deformation characteristics of negative Poisson's ratio honeycomb.

The main conclusions can be summarized as follows:

1. It is found that the corresponding deformation mode under static load loading in the y and x directions is arc crushing and layer by layer crushing. However, under the high-speed impact, the deformation mode in both directions is layer-by-layer crushing.
2. According to the deformation mode obtained by finite element simulation, the analytical formula of uniaxial crushing stress in two directions of RHA is derived by the two-scale method. The analytical solution of collapse stress is basically consistent with the finite element results. The second platform stress of the honeycomb is calculated. The second platform stress is much larger than the first platform stress. The honeycomb has a higher energy absorption effect in the second platform stage.
3. According to the conservation of momentum, the quasi-static analysis results are derived from dynamic impact, and the dynamic impact collapse stress is verified by finite element simulation. It is found that as the speed increases, the platform stress of the honeycomb structure also increases. When the speed is lower than 50 m/s, the second plateau period of the honeycomb under the impact is more obvious. When the speed is higher than 50 m/s, the second plateau period disappears.

The special honeycomb structure studied in this paper has two platform periods. The first plateau period is the compressive deformation of the first-order re-entrant honeycomb, and the second stage is the further compression of the horizontal edge. This special deformation mode provides better mechanical properties and provides a new idea for future research of honeycomb structures.

## Data Availability

The data that support the findings of this study are available upon reasonable request to the corresponding author.
